# Qualitative analysis of mothers’ perception related to the delivery of information regarding preterm births

**DOI:** 10.1186/s12884-024-06404-3

**Published:** 2024-04-12

**Authors:** Doriane Randriamboarison, Elisa Fustec, Isabelle Enderlé, Mathilde Yverneau, Karine Le Breton, Linda Lassel, Nadia Mazille-Orfanos, Patrick Pladys

**Affiliations:** 1 Department of Neonatology, University Hospital of Rennes, Rennes, 35000 France; 2 Department of Obstetrics and Gynecology, University Hospital of Rennes, Rennes, 35000 France; 3 Faculty of Medicine Rennes 1 University, Rennes, France

**Keywords:** Preterm birth, Counseling, Perception, Qualitative, Interviews

## Abstract

**Background:**

Preterm birth is a major health issue due to its potential outcomes and socioeconomic impact. Prenatal counseling is of major importance for parents because it is believed that the risk of preterm birth is associated with a higher parental mental burden. Nowadays in France, the content and delivery of antenatal counseling is based on personal experience since there is a lack of official guidelines. The goal of the study was to evaluate maternal perception of antenatal information delivered in the setting of preterm births.

**Methods:**

A qualitative study was performed using semi-structured individual interviews of 15 mothers with a child born > 26–34 GW. Data analysis was based on a constant comparative method.

**Results:**

Concerning prenatal counseling content, parents wanted to be informed of their role in the care of their preterm child more so than statistics that were not always considered relevant. Parents’ reactions to the announcement of the risk of a preterm birth was dominated by stupefaction, uncertainty and anxiety. When it comes to the setting of prenatal counseling, patients’ room was deemed an appropriate setting by parents and ideally the presence of a coparent was appreciated as it increased patients’ understanding. The physicians’ attitude during the counseling was considered appropriate and described as empathic and optimistic. The importance of support throughout the hospitalization in the form of other parents’ experiences, healthcare professionals and the possibility to preemptively visit the NICU was emphasized by participants. Delivery experience was dominated by a sense of uncertainty, and urgency. Some leads for improvement included additional support of information such as virtual NICU visit; participants also insisted on continuity of care and the multidisciplinary aspect of counseling (obstetrician, neonatologist, midwife, nurse, lactation consultant and psychologist).

**Conclusion:**

Highlighting parents’ expectations about prenatal counseling could lead to the establishment of overall general guidelines. However, some topics like the use of statistics and mentioning the risk of death underline the importance of a personalized information.

**Supplementary Information:**

The online version contains supplementary material available at 10.1186/s12884-024-06404-3.

## Introduction

According to the latest French National Prenatal Survey (NPS), the rate of preterm birth was 7% in 2021, which represents about 46,000 newborns [1].This rate has remained stable since 2016. Most of preterm deliveries happen between 32 and 36 GW (5.3%), and1.7% occurbetween 22 and 31 GW [1].

Preterm birth has long term effects and even though survival without neuromotor or sensory disabilities has improved in the last decades from 45.5% in 1997 to 62% in 2011 [2], ex-preterm infants present more neurodevelopmental complications and motor disorders such as cerebral palsy, cognitive disabilities, school learning disabilities [3, 4]. Considering all these outcomes and their socioeconomic impact,preterm birth represents a major health issue. Therefore, preventingpreterm labor and neonatal complications associated with a preterm birth is of utmost importance [2]. An integral part of high-risk pregnancy management is announcing to the parents that their pregnancy is no longer as they had envisioned it. It is the healthcare professional’s responsibility to make sure the patient understands all the information necessary to apprehend the medical course and to make informed decisions [5]. In this setting, communication in addition to bringing emotional support and conveying empathy also plays a legal role. This information is delivered during an antenatal consultation.

During the antenatal consultation, neonatologists focus on neonatal complications and how to manage them. This has been shown to be helpful for parents [6]. It contributes to psycho-social support, lowers risk of postpartum depression and mother-infant bonding disorder [7]. However, this new knowledge may also contribute to parents’ anxiety [8]. Parents’ needs and expectations regarding antenatal counseling are not always correctly understood by clinicians [9, 10]. Furthermore, this information is often delivered in a stressful environment, where there is a concomitant concern for the mother’s health. Information concerning obstetrical outcomes must also be provided including causes of preterm birth, treatments, and prognosis. In this setting, delivery becomes an abrupt and unanticipated event which can generate an important amount of stress. High-risk pregnancies and emergency deliveries are more at risk to generate posttraumatic stress disorder than regular pregnancies (18.5% versus 4%) [11].Neonatal outcomes are deeply connected to the obstetrical path and perinatal collaboration between neonatal and maternal caregivers improves families’ experience in all aspects of preterm birth [12]. Therefore, the way in which information is delivered in the antenatal period has a major impact on the parents’ experience throughout their subsequent path.

Nowadays in France, the content and delivery of antenatal counseling is based on personal experience since there is a lack of official guidelines. Most studies evaluating antenatal counseling are focusing on extreme preterm birth [13–16]. However, even though preterm infants born after 26 GW are at lower risk of adverse outcomes, they represent an important population in terms of prognosis. Moreover, these studies essentially focus on parents’ role in deciding between active support and palliative care [17], overlooking all other aspects of prenatal information. Most research has also been conducted from a neonatological point of view without the obstetricians’ input.

The aim of this qualitative study is to evaluate maternal perception of antenatal information delivery in the setting of preterm birth between 26 and 34 GW. Our goal is to improve our practice by bringing some insights on how to best counsel patients at risk of preterm birth and help them understand complex information [18].

## Methods

### Study context

The neonatal and obstetric departments of the university hospital of Rennes offer prenatal counseling and maternal care to all patients at risk of preterm delivery. Information delivered is based on healthcare professionals’ experience. Concerning neonatal antenatal counseling, interviews are conducted by a senior neonatologist as soon as possible after patient’s hospital admission. If the situation evolves or if patient asks for an update, follow-up consultations may be conducted by the neonatal physician. The obstetric team (senior obstetricians and neonatologists, residents and midwives) informs patients on obstetrical care and prognostication. All information given is adjusted on clinical context and patient’s history.

### Design

A qualitative study was performed. We followed Consolidated Criteria for Reporting Qualitative Studies (COREQ) guidelines [19]. Then we analyzed quantitative population’s characteristics.

### Participants

Mothers with a child born between 26 and 34 gestation weeks admitted to the NICU at the tertiary care university hospital of Rennes from January 2019 to April 2020 and discharged from the hospital at the time of inclusion were selected. The time lag between birth and interview ranged from 6 months post-discharge to a maximum of 18 months, in order to minimize memory bias We included in our study mothers who had been hospitalized in the level 3 high risk pregnancy unit of the University hospital of Rennes and received prenatal counseling from a neonatal attending physician. Some patients had their first medical care at another hospital and then were transferred to the hospital of Rennes before birth. Transferred patients were also included. Our exclusion criteria were children born before 26 GW, deceased children, deceased mother, patients under 18 years old, patients who did not speak fluently French, patients with cognitive disabilities, patients without contact information, patients who gave birth in another hospital and patients whose child (or one of the children in case of multiple pregnancy) was still hospitalized at the time of the study.

### Setting and sample

A physician was responsible for explaining the research project to potential participants and for sending an email newsletter describing the purpose and outline of the research. Mothers were invited to participate in a semi-structured interview. Participants responded to this invitation via email. Investigators who conducted the interviews informed participants, in the letter and then orally, about the aim of the study and their right to withdraw their participation at any time without giving any reason. Reminders were then sent via emails to the participants who did not respond to the letter. Patients who did not have an email address were recruited by phone calls made by one of the investigators. All mothers gave their informed consent before participating. We planned on stopping inclusion of patients when saturation was achieved (i.e. no new themes or ideas were generated by subsequent interviews).

Considering the difficulty for patients to come to the hospital for the interview, we initially let participants choose between a face-to-face interview or over the phone according to their convenience. In the face of the Sars-CoV-2 pandemic and its associated restrictions, all interviews were then conducted over the phone.

### Data collection

Semi-structured interviews were conducted in French by one or both interviewers who were a neonatal resident (DR) and an obstetrician-gynecologist (OB/GYN) resident (EF). Data collection spanned from June 2020 to March 2021. Interviews were semi-structured, with a predefined list of open-ended questions focusing first on the information received concerning the hospitalization, treatments, and prenatal counseling, and then on desired improvements, and open suggestions. The interview guide was developed by authors (DR, EF, NM, IE and KL) after a review of the literature before starting the study. If applicable, face-to-face interviews were conducted at a private office space located in the NICU.

**Table 1:** Standardized interview guide

**General information** In the course of your prenatal care, you may have received information about the risk of preterm birth. - Which interviews had the most impact on you in particular? For each interview, which professionals conducted them? - How did you feel about this information in general? How did you feel when the risk of preterm delivery was announced?
**About hospitalization** - How did you feel about receiving information about your hospitalization? Who provided this information? - What else would you have liked to know about your hospital stay? What do you remember about this information? What experience do you retain from this information?
**About treatments** - Which treatments do you remember receiving? How do you feel about the information regarding these treatments for managing your state of health (pre-eclampsia, etc.) and preterm birth? - What information have you missed about these treatments?
**Circumstances of the prenatal interview** - Where and when did this interview take place? With which healthcare professional? Were you accompanied during this interview (spouse, family member, etc.)? - What would you have liked for this antenatal interview (presence of someone in particular, with several professionals, at what point during hospitalization, etc.)?
**Content of the interview on preterm birth** - What important messages did you retain (figures, statistics, risk of neonatal death mentioned)? Did you miss any information or was it too detailed? - How did you feel about your interviewer’s attitude? What does the antenatal interview on preterm birth mean to you as part of your overall perinatal experience? - Which factors helped you to better apprehend this situation of preterm birth (close relatives or healthcare team’s support, experiences in the family, spirituality, etc.)? - How has your role with your child been described? - What additional resources would you have liked to have had available (written documents, videos, a visit to the neonatology department, etc.)?
**Points for improvement** - What information did you miss during this interview, in the light of your subsequent experience, and how could this interview be improved?

To ensure consistency, we used the same interview guide in every interview (Table 1). The interviewers received preliminary training on reformulation to carry out the in-depth interviews with qualitative method referents. They reported their involvement after each interview. Sessions were recorded with the consent of each participant and then transcribed verbatim and de-identified. The aims and rational for the research were disclosed to the participants in the newsletter. We confirmed patient’s understanding during the interview.

Throughout the session, the moderator summarized and reformulated the results and presented them back to the participants to ensure information was accurate and that their comments had been correctly understood. At the end of the session, participants completed a short quantitative questionnaire to obtain their socio-demographic characteristics. We obtained remaining socio-demographic data from the patient electronic medical record.

### Data analysis

The analysis procedure was conducted byfour researchers (EF, DR, NM and IE) using an inductive approach to identify themes that emerged from the data. Each transcript was independently read several times to facilitate immersion in the data.The thematic analysis of the data promoted a logic of emergence. The interviews were first analyzed using a manual method of coding the themes and sub-themes. The researchers used open coding process to summarize participants’ views by assigning words to quotes or paragraphs. The coding of the researchers were then compared and in the event of any discrepancies or a disagreement, other physicians (MY, KL, LL and PP) adjudicated. This method enhances the validity of the assigned themes. We kept including participants in the study until saturation was achieved (i.e. no new themes or ideas were generated by subsequent interviews).

The list of themes and sub-themes was then generated and extracted in tabular form. Constant comparative analysis was used to assess overall saturation [20]. Authors selected verbatim quotes to illustrate the thematic findings. We coded data from transcripts using the Saldaña method [21] To ensure the reliability of the coding and analysis of the data, findings were discussed among the authors. At the same time we used the NVivo® 12 Plus software interface (QSR International) to support the coding tree. The software was also used to check the frequency of occurrence of themes and to ensure that our main themes were consistent. NVivo’s contribution was also to facilitate the link between the highlighted themes and the verbatim references.

### Ethical considerations

The study was approved by the local Ethics Committee (reference number 20.61). Participation was on a voluntary basis. The university hospital of Rennes recorded the material in accordance with all French ethical regulations (ref: MR-003).

## Results

### Participants

We conducted a total of 15 interviews, which took place between June 2020 and March 2021.We obtained data saturation after 12 interviews. Amongst the three first participants who were given the choice of the interview setting, two of them decided on a face-to-face interview, and the last one over the phone. For all remaining participants, we only conducted phone interviews.Average length of interviews is 44 min ± 11 min (minimum 25 min, maximum 66 min). Face to face interviews lasted 32 and 42 min each.

Participant’s characteristics are presented in Table 2. On average, participating mothers were 31.4 years old (± 4.9 years). Newborns were on average 30.2 ± 2.5 weeks of gestation at birth.

**Table 2:** **Characteristics of participants** (*n* = 15 women; *n* = 18 babies)

Variables: n (%)						
**Age (years)**	18–25 26–35 36–45	2 9 4		(13) (60) (27)		
**Level of education**	Less than High School High School College ≤ 3 years College > 3 years	3 2 6 4		(20) (13) (40) (27)		
**Parity**	1 2	9 6		(60) (40)		
**Gestation**	Single Twins	12 3		(80) (20)		
**Length of hospital stay**	Less than 7 days Between 7 and 30 days Over 30 days	7 5 3		(47) (33) (20)		
**Mode of delivery**	Emergency caesarean Planned caesarean. Vaginal birth	11 2 2		(74) (13) (13)		
**Gestational age at delivery**	26–27 GW 28–30 GW 31–33 GW	3 3 9		(20) (20) (60)		
**Cause of preterm birth**	Pre-eclampsia Intrauterine growth restriction Placenta previa Premature rupture of membranes	6 5 2 2		(40) (33) (13) (13)		
**Neonatal complications in NICU**	Respiratory distress syndrome Heart failure Necrotizing enterocolitis Sepsis Intraventricular hemorrhage	16 1 4 5 3		(89) (5) (22) (28) (17)		
**Long-term complications linked to preterm birth**	Neurological Respiratory Eating disorder None	1 3 2 12		(5) (17) (10) (67)		

### Characteristics of prenatal counseling

Circumstances of prenatal counseling are reported in Table 3. Interviews mostly took place in the patient’s hospital room, and within the first days after admission.

**Table 3:** **Characteristics of prenatal consultation** (*n* = 15)

Variables: n (%)		
**Duration**	Less than 15 min Between 15 and 30 min Between 30 and 45 min More than 45 min	5 (33) 2 (13) 3 (20) 5 (33)
**Number of counseling interviews**	1 More than 1	11 (73) 4 (27)
**Localization**	Hospital room Delivery room	13 (87) 2 (13)
**Partner present during counseling**	Yes No	8 (53) 7 (47)
**Time between admission and first counseling**	Less than 48 h Between 3 and 7 days More than 7 days	11 (73) 3 (20) 1 (7)
**Time between last counseling and delivery**	Less than 48 h Between 3 and 7 days More than 7 days	4 (27) 9 (60) 2 (13)

### Thematic analysis

Seven themes were extracted from our data analysis. We subdivided each theme into sub-themes and illustrated some of them with participants’ quotes from the interview (presented in Table 4).

**Table 4:** **Themes extracted after data analysis** (in order of frequency)

Themes	Subthemes	Representative quotations
Prenatal counseling content	Neonatal complications and care	I was told that I would possibly not hear her [baby] cry but that it’s okay (Patient no 7) We were told there would surely be ups and downs through the hospitalization journey (Patient no 3)
	Parents’ role	When you are this early in your pregnancy, they [obstetricians] tend to still use the word ‘fetus’ *laughs* and it’s like a slap in the face, so when we are told to bring a cuddly toy, it makes it a little more real (Patient no 2)
	Use of statistics	We weren’t given any stats, which is a good thing because if we aren’t on the right side, we wonder ‘why us?’ (Patient no 2) We asked for a lot of figures. […] The first thing he [pediatrician] told us was ‘I can save babies that weight 500 grams’, […] and this was reassuring to hear (Patient no 8)
	Risk of death	I think she [obstetrician] was able to reason on a case-by-case basis and tell herself that I didn’t need that (Patient no 15) Of course, at 26 weeks it [risk of death] was mentioned, it’s not what I retained because at that moment, one wants to focus on the positive (Patient no 12) Because in any way, we wonder about it [risk of death] and get scared so might as well tell us directly (Patient no 3)
Mother’s feelings and reactions	Announcement of a risk of preterm birth	I wasn’t feeling sick and suddenly one day I was told I would stay in the hospital until I delivered (Patient no 4) I can’t explain it, but I didn’t have much concern about my baby. […] I had a feeling that everything would be all right (Patient no 6)
	Prenatal hospitalization experience	When everybody is gone, your family, your husband… it’s important to know that a nurse or someone else can come by if you are feeling down or worried (Patient no 3) All those medical rounds […], all those people […], every morning for 5 weeks […] it’s unbearable and stressful (Patient no 9) I remember very well seeing some doctors, but to tell you exactly what happened… It went so fast that I erased many details from my memory. All I knew was that I would have my belly cut open, that it would be a premature baby, but that’s it (Patient no 10)
Circumstances of prenatal counseling	Co-parent present	My husband and I noticed that when seeing the doctor, we didn’t retain the same pieces of information (Patient no 12)
	Organization of prenatal counseling	It happened in the hospital room and that was very nice, it’s an intimate place (Patient no 11)
	Counselor’s attitude	She [pediatrician] speaks with words which […] are not doctor words, she explains in our own lingo how things will go (Patient no 15) She [pediatrician] was both attentive and enlightening at the same time (Patient no 11)
Support during prenatal hospitalization	Close relatives	My husband came every day (Patient no 12) My family and my friends were there to support me (Patient no 14)
	Healthcare professionals	They were human, realistic but human, really sweet (Patient no 12)
	Shared experience with other parents	A friend of mine had experienced a severe premature birth, and it helped me a lot, it allowed me to have hope (Patient no 11)
	Tour / visit of the NICU	We make a big deal about it [NICU] in our minds, we feel like it’s morbid, like walls are dark […] and we get there, and we’re shocked because in fact no, there actually is life (Patient no 2) […] in order to understand the equipment, the different probs, the cables, what we could read on the scope and all of that. We already had the information, and it was useful for later (Patient no 3)
Delivery experience	Information on delivery	The hardest thing was… Well, no one could give me a date, tell me when it [delivery] would happen […] if it would be in two days or in three weeks (Patient no 3)
	Urgency of delivery	This was the moment I actually realized that there was a great chance that I would be cut open (Patient no 10) The day I delivered was in a rush, […] I could see everybody run, […] my husband wasn’t there, and I didn’t know if he would be able to come, it was […] very stressful (Patient no 1)
Additional sources of information	Written documents	I got a bunch of booklets, including on cesarean and breastfeeding (Patient no 1)
	Internet	I kept searching for pictures of premature babies, parents’ personal tales […] and I stumbled on very grim things (Patient no 8)
Suggestions for improvement	Additional support of information	Some pictures of […] what a premature baby looks like – because we imagine a big deal in our heads, and in the end, they are just babies, tiny, but still babies (Patient no 2) I believe that people don’t wish for the same things. Presenting a video to someone who doesn’t want to picture their preterm baby, can get their back up (Patient no 8)
	Antenatal information	They [pediatric nurses] have a different insight and way of explaining things compared to the pediatrician (Patient no 3) When in that situation, we wish to talk to people who are facing the same things (Patient no 11)
	Postpartum care	I was directly taken to my maternity bedroom, except my baby wasn’t there with me, and I could hear other babies’ cries every night. [It was] very troubling and traumatizing (Patient no 1)

1. Prenatal counseling content.
  - *Neonatal complications and care*.

Information delivered during prenatal counseling was the most mentioned during the interviews. Participants recalled being told about neonatal complications. They talked about respiratory outcomes first, short and long term. Then neurological complications were evoked including specific follow-up and neurosensorial risks. Mothers also reported receiving information concerning the NICU: the rooms, the equipment, the incubator. They remembered being told about the usual medical course and the steps during hospitalization.

b. *Parents’ role*.

How participants should act with their preterm newborn is commonly addressed during antenatal consultation. Mentioning the baby’s future life makes parenthood more real. For instance, practical aspects such as transferring parents’ smell through comfort blankets and cuddly toys were greatly appreciated. Being able to spend unlimited time with their child was also reassuring. Breastfeeding is another important topic, especially knowing that it is feasible even in case of preterm delivery. This notion was carried by the obstetric team. Midwives adapted their support to patients’ need, no matter what they first wished. A participant explained that she changed her mind based on the information she received about the role of breast milk for preterm babies: *‘Midwives who listened, who taught me how to pump my milk even though I was totally reluctant to breastfeed’* (patient no 2).

Finally, participants mentioned skin to skin as a beneficial act to their child’s well-being. Mothers report highly on it, as shown by patient no 8’s quote: *“they told me that I could stay close to her, that I was going to be able to touch her […] to hold her against me. When I was told that, I felt a lot better because I didn’t know I would have the opportunity to hold her.”*

c. *Use of statistics*.

To participants, statistics and numbers were either not mentioned or considered irrelevant. Indeed, seven patients reported not receiving any and seven had no recall of any statistics. Only one patient was looking for statistical data in the prenatal counseling and insisted on receiving some. When asked if they wished they were given some, four participants were against, four would have appreciated it and seven had no opinion. The ones in favor explained they wanted to hear positive numbers such as survival rates. Some participants described themselves as wanting to know everything and be as informed as possible. Participants who did not wish to receive any statistics argued that it would have scared them, and made them worry about worst case scenario.

d. *Risk of death*.

Mortality of preterm children was not mentioned to every participant as four participants reported death not being talked about during antenatal counseling. Avoiding this subject was appreciated by some participants. One mentioned they felt like practitioners could sense which information was relevant to them. To other participants, not talking about death could lead parents to imagine the worst-case scenario.

2) Mothers’ feelings and reactions.
  - *Announcement of a risk of preterm birth*.

Participants often reported feeling paradoxically in good health while being diagnosed with a risk of preterm birth. Therefore, such a diagnosis was reported as being a shock. Another feeling commonly mentioned is fear for the child’s health. On the contrary, some participants felt optimistic.

b. *Prenatal hospitalization experience*.

When asked how their hospital stay went, participants reported as many positive aspects as negative ones. They generally appreciated the close medical attention and support which were reassuring. However, some of them also mentioned the difficulty to accept the fact that they needed to stay in the hospital. Feelings mentioned by order of frequency were stupefaction, uncertainty, hope and anxiety. The sudden change during their pregnancy brought disorientation to some participants. Another feeling described was not knowing exactly what would happen to them and when delivery would occur. Participants also mentioned developing some hope during their hospital stay, especially for participants who were hospitalized for the longest period of time. As time went by and nothing serious was happening, they found themselves hoping they would slowly escape preterm birth’s adverse outcomes. The whole experience of a risk of preterm birth generated anxiety for several participants. They continuously feared for their child’s life. Moreover, being hospitalized, away from their homes and relatives, could enhance this anxiety.

3) Circumstances of prenatal counseling.
  - *Co-parent present*.

Both parents being present during antenatal counseling was the most frequent situation. Having the other parent present allowed to reflect further on what had just been said. It kept the information alive and encouraged questions.

b. *Organization of prenatal counseling*.

All participants could describe how prenatal counseling went. Consultations happened in their hospital room, which participants found appropriate.

c. *Counselor’s attitude*.

Participants commented on the physician’s skills. Fourteen of them defined the neonatologist as optimistic, and showing empathy. They reported the physician using understandable language to them. According to participants, the counselor also personalized information according to the patient and the situation, as Patient no 1 mentioned: *‘I think they really understood [me] and told me what I needed to know without telling me too much.’*

4) Support during prenatal hospitalization.

Close relatives seemed to be the most important emotional support throughout hospitalization. The other parent was the most mentioned, followed by first-degree family members, especially mothers and sisters, and for some participants, friends. Healthcare professionals were also referred as supportive. Midwives and assistant nurses were in the first line of patient’s care and mothers relied on them. The psychologist was also cited, bringing moral, psychological, and emotional support. Shared experience with other parents who went through a similar path were appreciated by participants. They mentioned feedback from relatives who had a preterm delivery, letters, and pictures from former parents of NICU’s babies, who are now doing well. One patient said she had the need to search the internet, even though it did not necessarily bring her comfort. The tour of the NICU was also appreciated by mothers and considered as a real source of support.

5) Delivery experience.

Participants described information on delivery as clear but mentioned the difficulty dealing with delivery’s unpredictability. They had questions on how far in their pregnancy they could possibly go, whether they were going to deliver vaginally or by cesarean, if they were going to be induced. Mothers also talked a lot about the urgency of delivery and reported a feeling of being rushed. The need for support in this difficult situation was important. The presence of the co-parent was requested by participants, although it may not always have been possible if delivery was impending. They counted on the midwives and the obstetric team to support them as well.

6) Additional sources of information.

The most mentioned source of information was the tour of the NICU, when the patient’s health allowed it, and delivery was not impending. Written documents were also presented to patients and appreciated. Most participants mentioned receiving paper documents, including one on breastfeeding and one explaining planned cesarean section. Some participants reported searching information on the internet.

7) Suggestions for improvement.
  - *Additional support of information*.

Participants suggested pictures and videos. A virtual tour of the NICU to show the rooms with their equipment was also mentioned. The expectation of what the photographs should describe was controversial. Pamphlets with pictures of staff members to help identify each professional’s face and tasks were suggested. Written documents about local neonatal units, from highly intensive care to current care, and how they connect to each other, would be appreciated as well. Explanations on milk collection centers (lactarium) were also requested as several participants did not have a complete understanding of their functioning.

b. *Antenatal information*.

Participants wanted the same practitioners to perform the consultation, as they sought continuity of care and commitment from healthcare professionals. Several participants also mentioned that the presence of a neonatal nurse during the neonatologist’s counseling would be beneficial. One participant suggested having the psychologist present to adjust psychological follow-up after the meeting. Sharing other parents’ experiences was also brought up. Participants wished they could have joined talk groups in the high pregnancy risks unit. Participant no 9 suggested to tell future parents confronted with a risk of preterm birth about the care of a preterm child: *“And to tell them it’s a fight for the baby and it’s a fight for the parents.”*

c. *Postpartum care*.

Several participants addressed postpartum mothers’ care. They expressed the need to be hospitalized in a unit without any newborn instead of the usual post-delivery maternity units, as it made the absence of their child harder to endure. Some of them even wished to be in the same room as their infant, included in the intensive care unit, such as Kangaroo Mother Care (KMC) units. Another commonly mentioned topic was breastfeeding: they wished for more help and support during the first steps of setting breastfeeding.

## Discussion

This study on the information related to preterm birth and its consequences, delivered during prenatal care, gives a thorough insight into the perception of mothers faced with the care of a preterm infant. The announcement of a risk ofpreterm birth came as a shock for patients, as there often was no forerunner. However, the information delivered byneonatologists was overall described as clear, adapted, and carried out with optimism and empathy. Concerning hospitalization in the high-risk pregnancy unit, participants emphasized the importance of having different sources of support to help them cope with anxiety and unpredictability. The feedback provided by participants to improve the delivery of information included the development of visual sources of information.

Providing information on a situation that cannot be predicted is a difficult task. Parents need to be aware that the ability to give an accurate prognosis before delivery remains limited [22]. Our study shows that some parents wish to have as much information as possible to be fully prepared, whereas others would like to only hear what is very necessary. Many studies on prenatal counseling have shown the importance of personalized information. Most of them focus on the field of extreme prematurity. However, Gaucher et al. demonstrated, in a preliminary qualitative study of 5 interviews [23], results comparable to our own on the content of patients’ expectations during this antenatal interview. This initial study was followed by a quantitative study [24] designed to verify their results on a larger scale using a quantitative method. This is one of the few studies which has focused on the maternal experience beyond extreme prematurity, but with a quantitative approach. Healthcare professionals must try to identify parents’ expectations and adapt their speech accordingly [18]. Culture and social background should also be taken into consideration, as well as level of understanding [25]. Personalization is probably the most important aspect and should be applied to all parts of antenatal care [26, 27](. We also found these results in our study, but our qualitative approach, which is relevant for assessing mothers’ experiences, provided additional data on the way in which parents wish to receive this information. Learning how to identify parents’ wishes should be a part of residents’ training as it is not an easy task. Moreover, delivering unwanted information can create the wrong environment and hinder the parents and healthcare providers relationship [13, 14]. Misunderstanding can generate miscommunication and dissatisfaction which can lead to suboptimal care [18]. What practicians think parents understood may not reflect what parents actually report being told [22].

In our findings, the wish for statistics and figures varies from one participant to another. Physicians may be confronted with the question of whether or not to share them. A study showed that some mothers, especially those with a high education level appreciated exact statistics more than general facts [6]. It brings us back to the idea of personalizing our counsel. Geurtzen et al. showed that parents’ choice on statistics was divided, and if given, these should be well explained [26]. However, a systematic review on parent communication needs during antenatal consultations found that parents wished for more than only quantitative data concerning mortality and morbidity. For instance, they expect information on their role [14]. So before giving statistical data, physicians should seek if parents want them and provide them in a way that is understandable and relevant to this individual situation.

In our study, physician’s skills and attitude are well remembered by mothers, suggesting the idea that if parents feel in a safe and trusting environment, they will be more willing to listen, understand and ask questions. Other studies found that in order to improve pedagogy, the speaker should be compassionate, empathic, honest, and caring [10]. Nevertheless, parents also expect truth and real outcomes and importantly, in words they can understand [14]. Our study shows that participants had a positive experience with well conducted antenatal counseling, even though the risk of neonatal death was brought up by the physician. As pointed in previous research work, truthful information, even when difficult, can be expected from physicians regarding prenatal information. Some hope should also be provided, however some physicians may fear giving false hope [16]. The timing of the information delivery is another aspect of prenatal counseling that also needs to be personalized. Too soon can be stressful if the patient is still accommodating to their new situation [18]. On the contrary, too late may increase mother’s stress. Uncertainty of the prognosis and the possible threat of sudden emergency delivery add difficulty to the timing of antenatal counseling.

Several participants from our study brought up the positive impact of a nurse being present during counseling, which illustrates the importance of multidisciplinarity. Indeed, it has been shown that nurses can rephrase and check parents’ understanding [14]. Moreover, in the Netherlands, guidelines mention antenatal counseling should be performed with both an obstetrician and aneonatologist [15]. An American study supports the idea that optimal communication between the obstetric and neonatal teams improves outcomes and safety during the peripartum period [28]. When combined with an obstetric expertise, neonatal information can be more accurate and adapted to the degree of emergency. .

The use of multiple means of information delivery was supported by our participants including written, oral and visual. A study on the use of a decision aid in antenatal counseling showed that written information was often too complicated and understanding relied on parents’ educational level. Consequently, written information should be completed by oral explanations from a professional [18]. This has proven its efficacy in the obstetric field [25]. Such documents should be preferably personalized and adjusted to parents’ needs [26]. A visual support can decrease mothers’ anxiety [7]. Indeed, the time between antenatal counseling and the actual day of delivery can be long and mothers’ memory of the information delivered may fade. Visual aid can help parents remember information, even more so in a stressful environment [29]. A. D. Muthusamy et al. [30] found that submission of the medium before or while the information is being delivered improved recall of the information and decreased anxiety. However, providing this support after the information has been delivered is not very effective. Written information may not improve factual recall after verbal counseling of mothers in preterm labor [31]. Concerning the support of written information, Nicole M Rau and al provided that a paper handout and multimedia tablet were equally effective in the labor unit to supplement verbal preterm birth counseling and decrease parental anxiety [32]. This approach could be used in the setting of antenatal counseling. Alongside official documents provided by the hospital, the use of the Internet as a means of information has become increasingly important for pregnant women over the years [28]. In our study, the internet was depicted as negative because mothers mostly reported on their “worst case scenario” findings. However, other research show that even though internet findings may generate anxiety, they can also reassure mothers-to-be and be a rich and accessible source of support [33].

## Strengths and limitations

Our study is novel as it explores the obstetric side, and the research team was multidisciplinary, including neonatologists, obstetricians, and a psychologist. Moreover, the fact that we did not focus on periviable terms enabled us to study several aspects of prenatal counseling other than decision-making. Even though our interviews were conducted over the phone for the most part due to the sanitary conditions, the interview durations were satisfactory which shows participants’ trust towards researchers. Furthermore, we included patients who gave birth at least 6 months before the interview, and whose child was discharged which gave participants time to process what happened, allowing them to tell their experience. Another asset of this study is that it reflects real world experience and not a simulation like many previous studies [34].

One of the limitations of our study is that our results are impacted by some mothers’ characteristics: our participants mostly had preeclampsia. In consequence, we cannot generalize our results to all high-risk pregnancy hospitalizations, in particular spontaneous preterm labor. However, preeclampsia causes longer hospital stays and thus allows deeper insights on the hospital experience. Patients who present with spontaneous preterm labor sometimes don’t have time to receive antenatal counseling before delivery. Other biases to consider are gestational age at admission and delivery, and pathology of the newborns, as they may have influenced participants’ experiences. We also did not include mothers who had lost their child. They probably have a very different insight that is important to consider. This stems from the fact that we decided to not include very extreme preterm children, therefore mortality was less important in our population. In order to explore mothers’ point of view after the loss of their child, the research team would have to be well prepared to deal with grief and bring emotional support during the study. Another population that was not included was mothers who had gotten prenatal counseling but ended up delivering at full term. We did not explore the impact of such information on preterm birth and the stress generated on those patients. Fish et al. showed that prenatal counseling improved parental knowledge and satisfaction without increasing anxiety [35]. Finally, in this study we focused on mothers’ experiences. It would be interesting to compare them with the coparents’ point of views, as there could be differences in psychosocial perceptions between them.

To improve the delivery of information related to preterm births, several leads could be explored. Using simulation to personalize the information in prenatal counseling remains interesting and has been widely described in the literature, but an evaluation of the clinical implementation after this simulation training is essential. Furthermore, multidisciplinarity could be developed by training different specialists to perform prenatal counseling. Written documents and videos may be elaborated to improve patients’ understanding.

## **Conclusion**

The risk of preterm birth is a complex situation and all involved healthcare professionals should reflect on the best way to inform and support patients. Providing some general guidelines on how to respond to mothers’ expectations could be relevant, however personalization is the most fundamental aspect to keep in mind when delivering information on preterm birth. Hence the skills associated with information delivery in preterm births could benefit from the development and improvement of tools like: healthcare professionals’ training, interview guide for physicians that integrates parents’ expectations, and multidisciplinary counseling including all actors involved in the care of the mother and the child.

### Electronic supplementary material

Below is the link to the electronic supplementary material.

Supplementary Material 1

## Data Availability

All authors had full access to the data and materials. Data is available from Nadia Mazille-Orfanos (nadia.mazille@chu-rennes.fr) upon reasonable request.
