# Supplementary material for: Qualitative analysis of mothers’ perception related to the delivery of information regarding preterm births
Source: BMC Pregnancy Childbirth. 2024 Apr 12;24:272. doi: 10.1186/s12884-024-06404-3 (PMC11015681; doi:10.1186/s12884-024-06404-3)
Supplement: Supplementary file 1 — Supplementary Material 1 [file 12884_2024_6404_MOESM1_ESM.docx]

**COREQ**

| **Domain 1: Research team and reflexivity** | | |
| --- | --- | --- |
| Personal Characteristics | | |
| 1. Interviewer/facilitator | Which author/s conducted the interview? | Doriane Randriamboarison and Elisa Fustec |
| 2. Credentials | What were the researcher’s credentials? *E.g. PhD, MD* | Doriane Randriamboarison: MD  Elisa Fustec: MD  Karine Le Breton: MD  Mathilde Yverneau: MD  Nadia Mazille-Orfanos : MD  Isabelle Enderle : MD  Linda Lassel: MD  Patrick Pladys: MD - PhD |
| 3. Occupation | What was their occupation at the time of the study? | Doriane Randriamboarison : pediatric resident  Elisa Fustec : obstetrician resident  Karine Le Breton: clinical psychologist  Mathilde Yverneau: neonatologist  Nadia Mazille-Orfanos : neonatologist  Isabelle Enderle : obstetrician  Linda Lassel: obstetrician  Patrick Pladys: neonatologist |
| 4. Gender | Was the researcher male or female? | Doriane Randriamboarison : female  Elisa Fustec: female  Karine Le Breton: female  Mathilde Yverneau: female  Nadia Mazille-Orfanos : female  Isabelle Enderle : female  Linda Lassel: female  Patrick Pladys: male |
| 5. Experience and training | What experience or training did the researcher have? | Doriane Randriamboarison: pediatric residency with participation in medical research protocols  Elisa Fustec: pediatric residency with participation in medical research protocols  Karine Le Breton: MD psychology degree with participation in medical research protocols  Mathilde Yverneau: MD with PhD gradation in progress  Nadia Mazille-Orfanos : board certified neonatologist with university degree in qualitative research  Isabelle Enderle :Md PhD  Linda Lassel: MD with many publications in clinical research  Patrick Pladys: board certified neonatologist with a habilitation to conduct research and experience in qualitative research |
| Relationship with participants | | |
| 6. Relationship established | Was a relationship established prior to study commencement? | No relationship before the start of the study |
| 7. Participant knowledge of the  interviewer | What did the participants know about the researcher? *e.g. personal goals, reasons for doing the research* | The participants knew that the research was conducted as an end of residency project by the pediatric residents which aim was to better understand how information was received by parents faced with a preterm birth. |
| 8. Interviewer characteristics | What characteristics were reported about the interviewer/facilitator? *e.g. Bias, assumptions, reasons and interests in the research topic* | The interviewers do not yet have experience of pre-natal interviews but have already attended these consultations. They are future professionals in this field. |
| **Domain 2: study design** | | |
| Theoretical framework | | |
| 9. Methodological orientation and Theory | What methodological orientation was stated to underpin the study? *e.g. grounded theory, discourse analysis, ethnography, phenomenology, content analysis* | Content analysis |
| Participant selection | | |
| 10. Sampling | How were participants selected? *e.g. purposive, convenience, consecutive, snowball* | Purposive |
| 11. Method of approach | How were participants approached? *e.g. face-to-face, telephone, mail, email* | Face-to-face and e-mail |
| 12. Sample size | How many participants were in the study? | 15 participants |
| 13. Non-participation | How many people refused to participate or dropped out? Reasons? | - 2 additional participants agreed to participate but were not included as saturation had already been reached - 2 participants refused to participate. The ethics committee protects participants by allowing participants to decline taking part in the study without having to state a reason. One of the participants reported lack of time as the reason. - 6 did not answer the phone |
| Setting | | |
| 14. Setting of data collection | Where was the data collected? *e.g. home, clinic, workplace* | One interview done at home.  All the others at workplace (CHU Rennes) |
| 15. Presence of non-participants | Was anyone else present besides the participants and researchers? | No |
| 16. Description of sample | What are the important characteristics of the sample? *e.g. demographic data, date* | The important characteristics are all listed in table 1 of the manuscript |
| Data collection | | |
| 17. Interview guide | Were questions, prompts, guides provided by the authors? Was it pilot tested? | The methodology team checked the appropriateness, as well as the intelligibility of the questions in two initial test sessions |
| 18. Repeat interviews | Were repeat interviews carried out? If yes, how many? | No |
| 19. Audio/visual recording? | Did the research use audio or visual recording to collect the data ? | Audio recording |
| 20. Field notes | Were field notes made during and/or after the interview or focus group? | No field notes. But an observer was present in addition to the moderator during the interviews in order to reformulate or repeat certain questions without taking notes |
| 21. Duration | What was the duration of the interviews or focus group? | Duration of the interviews : between 11 and 24 minutes |
| 22. Data saturation | Was data saturation discussed? | We carried out a constant and comparative analysis and discussed saturation on the basis of 12 interviews. We continued up to 15 interviews and chose not to interview 2 additional participants because saturation was obtained. |
| 23. Transcripts returned | Were transcripts returned to participants for comment and/or correction ? | No but throughout the session, the moderator summarized and reformulated the results and presented them back to the participants to ensure information was accurate and that their comments had been correctly understood. |
| **Domain 3: analysis and findings** | | |
| Data analysis | | |
| 24. Number of data coders | How many data coders coded the data? | 4 researchers |
| 25. Description of the coding tree | Did authors provide a description of the coding tree? | Yes |
| 26. Derivation of themes | Were themes identified in advance or derived from the data? | Derived from data.  Our analysis used an inductive method to highlight the emergence of themes. |
| 27. Software | What software, if applicable, was used to manage the data? | The interviews were analyzed using a manual method of coding themes and sub-themes. We used the NVivo® 12 Plus (QSR International) software interface to support the coding tree, frequency of occurrence analysis and verbatim references. |
| 28. Participant checking | Did participants provide feedback on the findings? | No |
| Reporting | | |
| 29. Quotations presented | Were participant quotations presented to illustrate the themes / findings? Was each quotation identified? *e.g. participant number* | Each of the citations was identified by its participation number, to which the characteristics in Table 1 of the manuscript refer. |
| 30. Data and findings consistent | Was there consistency between the data presented and the findings? | This study hypothesizes an internal methodological coherence between the object of the research and the thematic analysis of the data, promoting a logic of emergence. |
| 31. Clarity of major themes | Were major themes clearly presented in the findings? | Yes, the main themes most raised by interviewees are discussed in the manuscript. |
| 32. Clarity of minor themes | Is there a description of diverse cases or discussion of minor themes? | Our results highlight the logical emergence of themes and sub-themes by their frequency of appearance, thus addressing the minor themes at the end. |
